# Amlodipine attenuates oxidative stress in the heart and blood of high-cholesterol diet rabbits

**DOI:** 10.5830/CVJA-2010-091

**Published:** 2012-02

**Authors:** I Salehi, M Mohammadi, F Mirzaei, FG Soufi

**Affiliations:** Department of Physiology, Faculty of Medicine, Hamadan University of Medical Sciences, Hamadan, Iran; Drug Applied Research Center, Tabriz University of Medical Sciences, Tabriz, Iran; Drug Applied Research Center, Tabriz University of Medical Sciences, Tabriz, Iran; Lung and Tuberculosis Research Center, Tabriz University of Medical Sciences, Tabriz, Iran

**Keywords:** oxidation stress, cholesterol-fed rabbits, lipid peroxidation, amlodipine

## Abstract

**Introduction:**

Oxidative stress is a key component of atherosclerosis. It has been suggested that amlodipine inhibits oxidative stress. In this study, we evaluated the effects of amlodipine on the total antioxidant capacity of heart tissue and blood in 36 control and cholesterol-fed male New Zealand white rabbits.

**Methods:**

The rabbits were divided into four groups (*n* = 9). Group 1 rabbits were fed a regular diet, group 2 were fed a diet with 2% cholesterol, group 3 were fed a regular diet plus 5 mg/kg/day oral amlodipine, and group 4 were fed 2% cholesterol diet plus amlodipine 5 mg/kg/day. At the end of eight weeks, blood samples were drawn and at the same time heart tissue was isolated and frozen in liquid nitrogen. After homogenisation, the solution was centrifuged and the light supernatant was stored at –80˚C. This was used for determination of glutathione peroxidase (GPX), superoxide dismutase (SOD) and (MDA) levels.

**Results:**

Eight weeks of amlodipine treatment significantly reduced the levels of total cholesterol, low-density lipoprotein cholesterol and triglycerides in the group on the hypercholesterolaemic diet (*p* < 0.05). In the blood, the level of thiobarbituric acid-reactive substances increased in the rabbits on the 2% cholesterol diet (group 2) and 2% cholesterol-plusamlodipine diet (group 4) and decreased in the amlodipineonly group (group 3) (*p* < 0.05). Lipid peroxidation in the heart tissue was similar to that in the blood, except in the amlodipine-only group (group 3). In the blood, the activity of total SOD (tSOD) decreased in the group on the 2% cholesterol diet (group 2) (*p* < 0.05) and markedly increased in the amlodipine-only (group 3) and 2% cholesterol-plusamlodipine groups (group 4) (*p* < 0.05).

**Conclusion:**

Amlodipine decreased oxidative stress in the heart and blood and improved the lipid profile in cholesterolfed rabbits. Therefore, it may be considered a useful tool for the reduction of oxidative stress and improvement of lipid profiles in diseases related to atherosclerosis.

## Summary

Oxidative stress, an imbalance between the production of reactive oxygen species (ROS) and their detoxification by antioxidants, is involved in cardiovascular diseases such as atherosclerosis, hypertension and heart disease.^1^,^2^ ROS cause oxidation of membrane phospholipids, proteins and DNA,^3^ and result in contractile failure and apoptosis in cardiomyocytes.^1^,^4^ But short-term oxidative stress may also be important in the prevention of aging by the induction of a process called mitohormesis. ROS can also be beneficial as they are used by the immune system to attack and kill pathogens.^2^

Atherosclerosis represents a state of heightened oxidative stress characterised by lipid oxidation in the vascular wall.^5^ Overproduction of ROS under pathophysiological conditions is an important part of atherosclerosis. Therefore oxidative stress is considered to play a key role in the pathogenesis of atherosclerosis.^6^,^7^

It has been proposed that oxidative stress plays an important role in the inflammatory processes that are key components of atherosclerosis.^8^ Under physiological conditions, the toxic effects of ROS can be reduced by enzymatic and non-enzymatic antioxidants.^9^ Superoxide dismutase (SOD), glutathione peroxidase (GPX) and catalase (CAT) provide the first line of enzymatic antioxidant defence against ROS-mediated cardiac injury.^1^,^10^

To improve the prognosis of patients with heart disease and injury, novel therapeutic strategies have focused on regulating oxidative stress in the cardiovascular system. Since both oxidative stress and inflammation need the participation of calcium ions (Ca^2+^) to cause atherosclerosis, calcium channel blockers (CCBs) are widely used in the cardiovascular field for the control of angina and hypertension and as an alternative to β-blockers in patients with heart failure.^11^,^12^

Amlodipine, a third-generation dihydropyridine CCB, has a high affinity for the lipid constituents of the cellular membrane. There is much basic and clinical data indicating that amlodipine, in addition to having haemodynamic properties, exerts noncalcium channel-related modulation in the vasculature, such as antioxidant activity.^13^ Since the antioxidant activity of amlodipine, particularly in the heart, has not yet been well examined, we aimed to investigate the role of amlodipine in the heart and blood of rabbits.

## Methods

Thirty-six male New Zealand white rabbits (± 1.4 kg) were obtained from the laboratory animal house of Tabriz University of Medical Sciences. They were housed in an animal room at 22–24ºC and given free access to commercial rat chow and tap water. All the experimental procedures used, as well as rabbit care and handling were in accordance with guidelines provided by the experimental animal laboratory and approved by the Animal Care Committee of the Tabriz University of Medical Sciences.

The rabbits were equally divided (*n* = 9) into four groups: group 1 rabbits were fed a regular diet, group 2 were fed a diet containing 2% cholesterol, group 3 had a regular diet plus 5 mg/ kg/day oral amlodipine, and group 4 had a diet with 2% choles- terol plus amlodipine 5 mg/kg/day, for eight weeks. Cholesterol powder and amlodipine powder were provided from Merck and Aria companies, respectively. Cholesterol powder was mixed into the feed. Amlodipine was dissolved in distilled water and was given with a special gavage tube at 09:00 daily for eight weeks.

The study protocol was designed in accordance with the *Guidelines for the Care and Use of Laboratory Animals* published by the US National Institutes of Health (NIH Publication, No. 86-23, revised 1996) and approved by the Ethics Committee for the Use of Animals in Research at Tabriz University of Medical Sciences.

At the end of the experiments, all animals were fasted for eight hours and then anesthetised by injecting ketamine (25 mg/kg, intravenously) and sodium pentobarbital (20 mg/kg, intravenously) via the ear vein. Blood samples were drawn from the inferior vena cava and were stored in tubes for determination of serum lipid profiles and blood oxidative stress.

After decapitation, the heart was quickly removed, washed in ice-cold saline and the atria and great blood vessels were trimmed away. The ventricles were weighed and quickly frozen in liquid nitrogen.

For analysis of oxidative stress, cardiac and blood homogenates were prepared at 0–4ºC as described by Rothermel *et al*.^14^ In brief, 50 mg of ventricle muscle were homogenised on ice in 1 ml of ice-cold lyses buffer (10 mM NaCl, 1.5 mM MgCl_2_, 20 mM HEPES, 20% glycerol, 0.1% Triton X-100, 1 mM dithi- othreitol, pH 7.4). The homogenates were centrifuged at 4 500 g for 1 min at 4°C (Avanti J 25 USA). The supernatant containing the cytoplasmic protein fraction was collected and a protease inhibitor cocktail (104 mM AEBSF, 0.08 mM aprotinin, 2 mM leupeptin, 4 mM bestatin, 1.5 mM pepstatin A, and 1.4 mM E-64) (P8340, Sigma-Aldrich, St Louis, MO) was added, and it was stored at –80ºC until use. Protein concentration of the super- natant was estimated using the Bradford technique.^15^

Blood samples were drawn from the inferior vena cava and stored in tubes for an hour. The serum was prepared and used for the determination of serum lipid profiles, including total cholesterol and triglycerides. These were determined by enzymatic methods using an automatic analyser (Abbott, Alcyon^300^ Falcor, USA).

Lipid peroxidation was analysed by measuring thiobarbituric acid-reactive substances (TBARs) in the homogenates, as previously described by Draper and Hadley.^16^ Briefly, the samples were mixed with 1 ml 10% trichloroacetic acid (TCA) and 1 ml 0.67% thiobarbituric acid. The samples were heated in a boiling water bath for 15 min, and butanol (2:1 v:v) was added to the solution. After centrifugation at 800 *g* for 5 min (Avanti J 25 USA), TBAR levels were determined from the absorbance at 535 nm.

SOD activity was determined using a RANSOD kit (Randox labs. Crumlin, UK), according to Delmas-Beauvieux *et al*.^17^ SOD activity was measured in the supernatant on a spectrophotometer (Stat Fax, 2100, Awareness, USA) at 505 nm. In this method, xanthin and xanthin oxidase were used to generate superoxide radicals, which react with 2-(4-iodophenyl)-3-(4-nitrophenol)-5-phenyl tetrazolium chloride (ITN) to form a red formazan dye. Concentrations of substrates were 0.05 mmol/l for xanthin and 0.025 mmol/l for INT. SOD activity was measured by the degree of inhibition of this reaction. To assay the mitochondrial SOD (mtSOD) activity in the heart, the cytosolic SOD was inhibited with 1 mm KCN.

Glutathione peroxidase activity was determined using a RANSEL kit (Randox labs Crumlin, UK) according to the method of Paglia and Valentine.^18^ GPX catalyses the oxidation of glutathione (at a concentration of 4 mmol/l) by cumene hydroperoxide. In the presence of glutathione reductase (at a concentration ≥ 0.5 units/l) and 0.28 mmol/l of NADPH, the oxidised glutathione is immediately converted to the reduced form with a concomitant oxidation of NADPH to NAD^+^. The decrease in absorbance at 340 nm was measured using a spectrophotometer.

## Statistical analysis

All determinations were performed at least in duplicate. Data were expressed as mean ± SEM and were analysed by a oneway ANOVA using a standard computerised statistical program, SPSS13.0 for windows software (SPSS INC, Chicago, IL, USA). When a significant *p*-value was obtained, the Tukey *post hoc* test was used to determine the differences between groups. A level of *p* < 0.05 was selected to indicate statistical significance.

## Results

Our results clearly demonstrate that eight weeks on a 2% highcholesterol diet significantly increased serum levels of total cholesterol (TC), low-density lipoprotein cholesterol (LDL-C), high-density lipoprotein cholesterol (HDL-C) and triglycerides (TG). These observations indicate that an atherogenic diet induced hypercholesterolaemia in our *in vitro* model. Although amlodipine treatment tended to enhance HDL-C:LDL-C and HDL-C:TC ratios in this group, these effects were not statistically significant. The significant increase observed in plasma levels of HDL-C and decrease in LDL-C, TG and TC is considered the main effect of amlodipine treatment on serum lipid profiles in the rabbits fed a regular diet (Table 1).

**Table 1:** Comparison Of The Serum Lipid Profile Changes (Mg/Dl)

*Variable*	*Group 1*	*Group 2*	*Group 3*	*Group 4*
Total cholesterol	49.13 ± 0.6	860.3 ± 0.6*	40.3 ± 0.8	524.5 ± 5.8*^#^
LDL	7.23 ± 1.39	722 ± 0.86*	13.13 ± 0.20	451.43 ± 6.70*^#^
HDL	14 ± 0.73	49 ± 0.63*	19.83 ± 0.54*	48.33 ± 0.95*
TG	95.50 ± 1.7	466.6 ± 2.5*	81 ± 0.50*	138.6 ± 1.8*^#^
HDL:LDL	2.47 ± 0.60	0.07 ± 0.001*	1.50 ± 0.05	0.11 ± 0.002*
HDL:TC	0.35 ± 0.02	0.06 ± 0.001*	0.4 ± 0.007*	0.09 ± 0.001*

Rabbits were fed a regular diet (group 1), 2% cholesterol diet (group 2), regular diet plus amlodipine 5 mg/kg/day (group 3) and a 2% cholesterol-plus-amlodipine diet (group 4). Data are expressed as mean ± SEM for each group (*n* = 9). Differences of *p* < 0.05 were considered significant. *Group 1 vs groups 2, 3 and 4; ^#^group 2 vs group 4.

## Lipid peroxidation

**Fig. 1:**
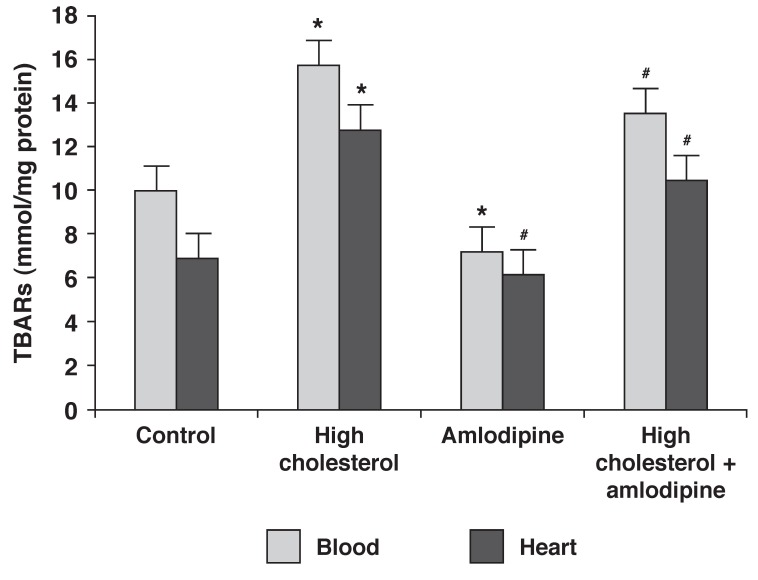
Effect of amlodipine and a high-cholesterol diet on TBAR levels. Data are expressed as mean ± SEM (*n* = 9) for each group. *p < 0.05 compared with control group, ^#^*p* < 0.05 compared with group 2.

In the blood samples, the level of TBARs increased in the rabbits on the 2% cholesterol diet (group 2) and those on 2% cholesterol plus amlodipine (group 4), and decreased in the group on a regular diet plus amlodipine (group 3) (*p* < 0.05). In addition, in those on a regular diet plus amlodipine (group 3) and those on 2% cholesterol plus amlodipine (group 4), the level of TBARs was less than in the group on a diet of 2% cholesterol (group 2) (Fig. 1). Lipid peroxidation levels in all the heart samples showed a similar trend to that of the blood samples, except in the rabbits on a regular diet plus amlodipine (group 3), where the levels of TBARs did not diminish compared to the control group (Fig. 1).

## Antioxidant enzymes

**Fig. 2:**
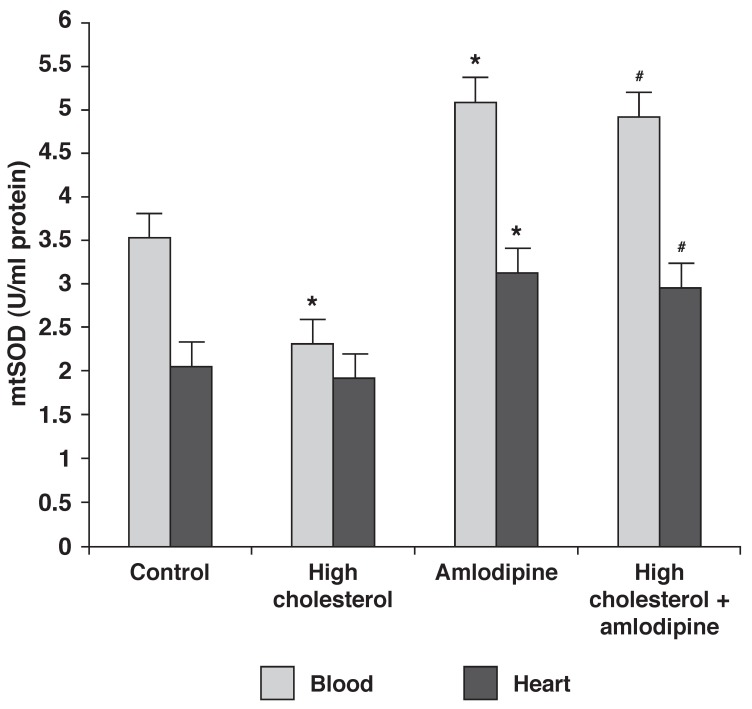
Effect of amlodipine and a high-cholesterol diet on SOD activity. Data are expressed as mean ± SEM (*n* = 9) for each group. *p < 0.05 compared with control group, ^#^*p* < 0.05 compared with group 2.

Fig. 2 shows that the activity of total SOD (tSOD) in the blood samples decreased in the rabbits on the 2% cholesterol diet (group 2) (*p* < 0.05), and markedly increased in those on a regular diet plus amlodipine (group 3) and those on 2% cholesterol plus amlodipine (group 4), compared with the controls (*p* < 0.05). Moreover, the activity of mitochondrial SOD (mtSOD) in the heart samples was enhanced in the rabbits on a regular diet plus amlodipine (group 3) and those on 2% cholesterol plus amlodipine (group 4), compared to the controls (group 1) and the 2% cholesterol group (group 2) (*p* < 0.05).

**Fig. 3:**
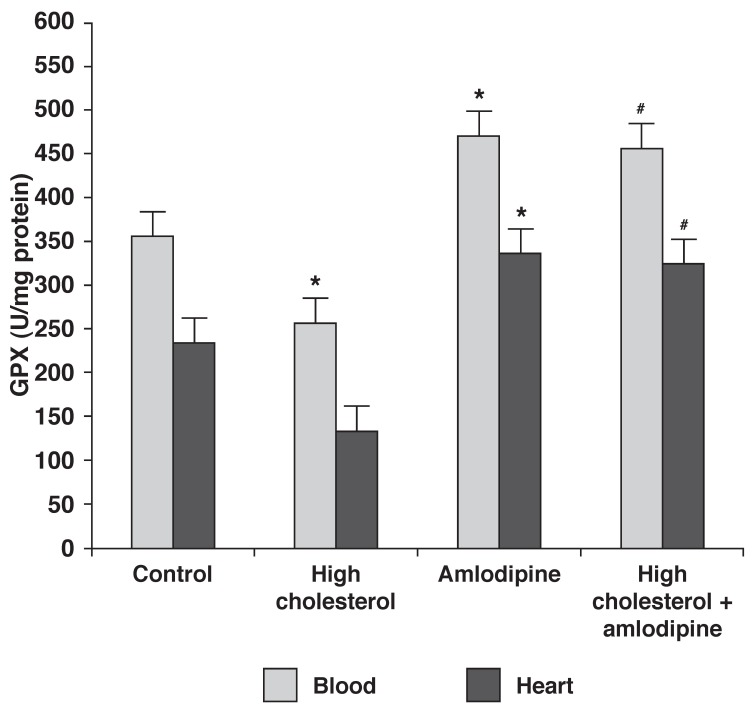
Effect of amlodipine and a high-cholesterol diet on GPX activity. Data are expressed as mean ± SEM (*n* = 9) for each group. *p < 0.05 compared with control group, ^#^*p* < 0.05 compared with group 2.

The changes in GPX activity (Fig. 3) in the heart samples were similar to the changes in SOD activity, except for the rabbits on the 2% cholesterol diet (group 2), whose GPX activity decreased compared to the control group (*p* < 0.05).

## Discussion

Our results indicate that eight weeks of a 2% high-cholesterol diet increased all serum cholesterol profile fractions and induced oxidative stress in the blood and heart tissue, since the level of TBARs (a marker of lipid peroxidation) significantly increased in all cholesterol-fed rabbits. The key finding of this study was that eight weeks of amlodipine treatment reduced oxidative stress in the blood and hearts of cholesterol-fed rabbits.

As an index of the anti-oxidative defence system, we measured the activities of SOD and GPX and the levels of MDA. A considerable number of studies have accumulated to suggest that most CCBs may be effective in preventing the development of atherosclerosis. The most important mechanisms involved may include antioxidant effects, and changes in Ca^2+^ and cholesterol metabolism.^19^-^21^

In this study, chronic amlodipine treatment increased HDL-C levels or the ratio of HDL-C to LDL-C, and reduced LDL-C and TG levels in the plasma. The importance of LDL-C, HDL-C and TC is well documented in the pathogenesis of atherosclerosis, but TG levels should not be ignored.^22^ In addition to the pivotal role that HDL-C plays in reverse cholesterol transport and cellular cholesterol efflux, it possesses a spectrum of anti-inflamma-tory, antioxidative, anti-apoptotic, antithrombotic, vasodilatory and anti-infectious properties, all of which potentially contribute to its atheroprotective nature.^23^ Accumulating evidence shows that HDL-C protects LDL-C from oxidation.

Epidemiological and observational studies have demonstrated that HDL-C level is a strong, independent predictor of risk of coronary heart disease (CHD), and raising HDL-C levels may afford clinical benefit.^24^ Since decreased HDL-C levels are associated with increased production of ROS, increases in HDL-C levels by amlodipine treatment may be a consequence of reduction in oxidative stress.^25^

MDA is one of the most reliable and widely used indices of oxidative stress and, as a marker of oxidative damage, has been studied extensively.^26^ It is known that lipid peroxidation is the co-operative event involved in the development of atherosclerosis.^27^ Also a positive correlation has been found between MDA levels and coronary artery calcification scores, and it is more convincingly correlated with TG levels and inversely correlated with HDL-C levels.^28^

In our study, as the level of MDA significantly increased in cholesterol-fed rabbits, amlodipine treatment reduced it in the blood and heart muscle. It has been suggested that highly lipophilic CCBs such as amlodipine, by inserting to a location in the membrane near conjugated double bonds, are capable of donating protons to lipid peroxide molecules, thereby blocking the peroxidation process.^21^ Since amlodipine is lipophylic, it easily enters the cells and prevents lipid peroxidation.^29^ In our results, increased MDA levels may have been attributed to a high production of free radicals by a high-cholesterol diet, and the ability of amlodipine to diminish lipid perxidation in the rabbits fed a high-cholesterol diet may have been non-calcium dependent and more related to proton donation.^30^

It has been proposed that a high-cholesterol diet induces free radical production and may result in oxidative stress, which plays an important role in hypercholesterolaemia-induced atheroschelerosis.^31^ Our data showed that a high-cholesterol diet decreased antioxidant enzyme activity (SOD and GPX) in both blood and heart tissue, and also confirmed that amlodipine could decrease the activation of oxidative stress. Previous reports indicate that markers of oxidative stress, such as superoxide production, were increased in atherosclerotic lesions.^32^ This antioxidant activity of amlodipine has also been observed in various animal models, including non-human primates, which reveals the important anti-atherogenic effect of this compound.^33^

Increased ROS production can initiate a cascade of signal transduction, which leads to endothelial dysfunction, changes in vascular tone, vascular remodelling and vascular inflammatory responses.^34^-^36^ Under normal conditions, the heart minimises oxidative stress-induced injuries by the enhancement of SOD and GPX activity. SOD converts superoxide radicals to H_2_O_2_ and GPX eliminates H_2_O_2_.^1^ Since hypercholesterolaemia significantly decreased GPX activity in the present study, this probably resulted in aggregation of H_2_O_2_ and other reactive oxygen species and may have caused lipid peroxidation. It has also been reported that amlodipine reduced oxidative stress by restoring copper/zinc-containing SOD activity in the heart in hypertensive rats.^34^

Amlodipine with or without a high-cholesterol (2%) diet significantly increased SOD and GPX activity in the heart and blood, compared with control or high-cholesterol fed groups. Surprisingly the activity of these antioxidant enzymes was approximately equal in groups 3 and 4. This suggests that the high-cholesterol diet could not decrease the antioxidant enzyme activity in the presence of amlodipine. Reduction of oxidative stress protects not only lipids, but also DNA, which is crucial to eventual cell death.^37^

Exactly how amlodipine exercises its antioxidant activity is unclear, although several possible mechanisms have been proposed, including antithrombotic, anti-inflammatory and antioxidant properties of HDL-C, and decrease in plasma LDL-C levels. The anti-oxidative property of these L-type CCBs may stem from their chemical structure; they contain an aromatic ring, which attracts free radicals. Furthermore, the dihydropyridine ring in these CCBs is able to donate a proton, which stabilises the free electron.^38^

Since CCBs primarily affect the cellular interaction of endothelial cells, smooth muscle cells, monocytes and thrombocytes, which play key roles in the early phases of the development of atherosclerosis, the amlodipine effect of inhibiting calcium influx is the main mechanism for attenuation of oxidative stress in atherosclerosis. Therefore one of the major pathways by which amlodipine exerts its antioxidant effect is prevention of calcium overload.^39^ There are some conflicting results in the literature that may be partly due to differences in *in vitro* models or the interventional drugs.^40^

## Conclusion

The present study indicated that the CCB amlodipine decreased oxidative stress in the heart and blood and improved lipid profiles in cholesterol-fed rabbits. It may therefore be beneficial for the reduction of oxidative stress and improvement of lipid profiles in patients with diseases related to hyperlipidaemia. Further clinical trials are needed to prove the importance of amlodipine and other CCBs in patients with atherosclerosis and similar diseases.

## References

[1] Tsutsui H (2006). Mitochondrial oxidative stress and heart failure.. Intern Med.

[2] Seddon M, Looi YH, Shah AM (2007). Oxidative stress and redox signalling in cardiac hypertrophy and heart failure.. Heart.

[3] McCord JM (1985). Oxygen-derived free radicals in postischemic tissue injury.. N Engl J Med.

[4] Siwik DA, Tzortzis JD, Pimental DR (1999). Inhibition of copperzinc superoxide dismutase induces cell growth, hypertrophic phenotype, and apoptosis in neonatal rat cardiac myocytes in vitro.. Circ Res.

[5] Ogunro PS, Balogun WO, Fadero FF, Idogun ES, Oninla SO, Elemile PO, Eziyi AK (2009). Plasma lipid peroxidation and totalantioxidant status among dyslipidaemic and hypertensive Nigerians with high risk of coronary heart disease.. West Afr J Med.

[6] Real JT, Martinez-Hervas S, Tormos MC (2009). Increased oxidative stress levels and normal antioxidant enzyme activity in circulating mononuclear cells from patients of familial hypercholesterolemia.. Curr Pharm Des.

[7] Nieman B, Rohrbach S, Catar RA, Muller G, Barton M, Morawietz H (2005). Native and oxidized LDL stimulate endothelin converting enzyme-1 expression in human endothelial cells.. Biochem Biophys Res Commun.

[8] Stocker R, Keaney JF (2004). Role of oxidative modification in atherosclerosis.. Physiol Rev.

[9] Ascensao A, Ferreira R, Magalhaes J (2007). Exercise-induced cardioprotection – biochemical, morphological and functional evidence in whole tissue and isolated mitochondria.. Int J Cardiol.

[10] Powers SK, Quindry JC, Kavazis AN (2008). Exercise-induced cardioprotection against myocardial ischemia-reperfusion injury.. Free Radic Biol Med.

[11] Okuda N, Hayashi T, Mori T (2005). Nifedipine enhances the cardioprotective effect of an angiotensin-II receptor blocker in an experimental animal model of heart failure.. Hypertens Res.

[12] Packer M, O’Conner CM, Ghali JK (1996). Effect of amlodipine on morbidity and mortality in severe chronic heart failure.. N Engl J Med.

[13] Hasegawa H, Takano H, Kohro T (2006). Amelioration of hypertensive heart failure by amlodipine may occur via antioxidative effects.. Hypertens Res.

[14] Rothermel  B, Vega RB, Yang J, Wu H, Bassel-Duby R, Williams RS (2000). A protein encoded within the Down syndrome critical region is enriched in striated muscles and inhibits calcineurin signaling.. J Biol Chem.

[15] Bradford MM (1979). A rapid and sensitive method for the quantitation of microgram quantities of protein utilizing the principle of protein-dye binding.. Anal Biochem.

[16] Draper HH, Hadley M (1990). Malondialdeyde determination as an index of lipid peroxidation.. Meth Enzym.

[17] Delmas-Beauvieux MC, Peuchant E, Dumon MF, Receuver MC, Le Bras M, Clerc M (1995). Relationship between red cell antioxidant enzymatic system status and lipid peroxidation during the acute phase of malaria.. Clin Biochem.

[18] Paglia DE, Valentine WN (1967). Studies on the quantitative and qualitative characterization of erythrocyte glutathione peroxidase.. J Lab Clin Med.

[19] Chong TZ, Yang SP, Pei D (2002). Amlodipine inhibits pro-inflammation cytokines and free radical production and inducible nitric oxide synthase expression in lipopolysaccharide/interferon γ stimulated cultured vascular smooth muscle cells.. JJP.

[20] Mancini GB (2002). Antiatherosclerotic effects of calcium channel blockers.. Prog Cardiovasc Dis.

[21] Mason RP, Marche P, Hintze TH (2003). Novel vascular biology of thirdgeneration L-type calcium channel antagonists: ancillary actions of amlodipine.. Arterioscler Thromb Vasc Biol.

[22] Pereira B, Costa Rosa Lf, Safi DA, Medeiros MH, Curi R (1994). Superoxide dismutase, catalase, and glutathione peroxidase activities in muscle and lymphoid organs of sedentary and exercise-trained rats.. Physiol Behav.

[23] Sharrett AR, Ballantyne CM, Coady SA (2001). Coronary heart disease prediction from lipoprotein cholesterol levels, triglycerides, lipoprotein(a), apolipoproteins A-I and B, and HDL density subfractions: the Atherosclerosis Risk in Communities (ARIC) Study.. Circulation.

[24] Wang M, Briggs MR (2004). HDL: the metabolism, function, and therapeutic importance.. Chem Rev.

[25] Ohara Y, Peterson TE, Harrison DG (1993). Hypercholesterolemia increases endothelial superoxide anion production.. J Clin Invest.

[26] Esterbauer H, Schaur RJ, Zollner H (1991). Chemistry and biochemistry of 4-hydroxynonenal, malondialdehyde and related aldehydes.. Free Radical Biol Med.

[27] Kutuk O, Basaga H (2003). Inflammation meets oxidation: NF- B as a mediator of initial lesion development in atherosclerosis.. Trends Mol Med.

[28] Jung HH, Choi DH, Lee SH (2004). Serum malondialdehyde and coronary artery disease in hemodialysis patients.. Am J Nephrol.

[29] Mogi M, Iwai M, Chen R (2006). Amlodipine treatment reduces stroke size in apolipoprotein E-deficient mice.. Am J Hypertens.

[30] Umemoto S, Kawahara S, Hashimoto R (2006). Different effects of amlodipine and enalapril on the mitogen-activated protein kinase/extracellular signal-regulated kinase pathway for induction of vascular smooth muscle cell differentiation in vivo.. Hypertens Res.

[31] Meilhac O, Ramachandran S, Chiang K, Santanam N, Parthasarathy S (2001). Role of arterial wall antioxidant defense in beneficial effects of exercise on atherosclerosis in mice.. Arterioscler Thromb Vasc Biol.

[32] Barry-Lane PA, Patterson C, van der Merwe M (2001). p47phox is required for atherosclerotic lesion progression in ApoE−/−mice.. J Clin Invest.

[33] Lundy A, Lutfi N, Beckey C (2009). Review of nifedipine GITS in the treatment of high risk patients with coronary artery disease and hypertension.. Vasc Health Risk Manag.

[34] Umemoto S, Tanaka M, Kawahara S (2004). Calcium antagonist reduces oxidative stress by upregulating Cu/Zn superoxide dismutase in stroke-prone spontaneously hypertensive rats.. Hypertens Res.

[35] Ushio-Fukai M, Alexander RW (2004; 264: 85–97). Reactive oxygen species as mediators of angiogenesis signaling: role of NADPH oxidase.. Mol Cell Biochem.

[36] Madamanchi NR, Vendrov A, Runge MS (2005). Oxidative stress and vascular disease.. Arterioscler Thromb Vasc Biol.

[37] Koyama Y, Takeishi Y, Takahashi H (2007). Azelnidipine inhibits H_2_O_2_-induced cell death in neonatal rat cardiomyocytes.. Cardiovasc Drugs Ther.

[38] Masumoto K, Takeyasu A, Oizumi K, Kobayashi T (1995). Studies of novel 1,4-dihydropyridine Ca antagonist CS-905. I. Measurement of partition coefficient (log P) by high performance liquid chromatography (HPLC).. Yakugaku Zasshi.

[39] Tanaka T, Nangaku M, Miyata T (2004). Blockade of calcium influx through L-type calcium channels attenuates mitochondrial injury and apoptosis in hypoxic renal tubular cells.. J Am Soc Nephrol.

[40] Candido R, Allen TJ, Lassila M (2004). Irbesartan but not amlodipine suppresses diabetes-associated atherosclerosis. Circulation.

